# Prognostic role of snail in lung cancer: Protocol for a systematic review

**DOI:** 10.1097/MD.0000000000011539

**Published:** 2018-07-13

**Authors:** Meng Li, Xing Zhang, Kaiwen Hu, Meiling Shi, Guangtong Dong, Daorui Li, Peitong Zhang

**Affiliations:** a Beijing University of Chinese Medicine; b Department of Oncology, Guang’anmen Hospital, China Academy of Chinese Medical Sciences; c Department of Oncology, Dongfang Hospital, Beijing University of Chinese Medicine, Beijing, China.

**Keywords:** lung cancer, prognosis, protocol, snail, systematic review

## Abstract

**Background:**

Increasing studies were performed to explore the prognostic value of snail in lung cancer (LC), however, with inconsistent results. Hence, this systematic review is aimed to evaluate the prognostic role of snail in patients with LC.

**Methods:**

A comprehensive literature search in the PubMed, Embase, the Cochrane Library, and Web of Science databases will be conducted to identify eligible studies. Language is limited as English. We will employ hazard ratios (HRs) and 95% confidence intervals (95% CIs) to estimate the correlations between snail expression and overall survival (OS), progression-free survival (PFS), recurrence-free survival (RFS), disease-free survival (DFS), and clinicopathological features. Meta-analysis will be performed using STATA 14.0 software.

**Results:**

This study will provide a high-quality synthesis of current evidence of the correlations between snail expression and OS, PFS/RFS/DFS, and clinicopathological features.

**Conclusion:**

The study will provide updated evidence to assess whether the expression of snail is in association with poor prognosis in patients with LC.

**Ethics and dissemination:**

It is not necessary for ethical approval because individuals cannot be identified. The protocol will be disseminated in a peer-reviewed journal or presented at a relevant conference.

**PROSPERO registration number:**

This systematic review protocol has been registered in the PROSPERO network (No. CRD42018095191).

## Abstract 2

Supplemental Digital Content is available in the text

## 1 Introduction

Lung cancer (LC) is currently a leading cause of cancer-related mortality.^[1]^ Histologically, it can be classified as non-small-cell lung cancer (NSCLC) and small-cell lung cancer (SCLC)—the former mainly consists of adenocarcinoma, squamous cell carcinoma, and large cell carcinoma and represents 85% of all cases of LC.^[2]^ Advances for LC have been slow, for which the 5-year relative survival is currently 18%. While more than one-half of cases are diagnosed at a distant stage, leading to an extremely poor 5-year survival rate of 4% among patients with stage IV LC.^[3]^ The high recurrence and metastasis largely contribute to the poor overall prognosis.^[4]^ Therefore, it is crucial to identify reliable therapeutic and prognosis biomarkers for LC.

The prognosis is highly associated with the metastatic behavior of the tumor.^[5]^ The epithelial to mesenchymal transition (EMT) seems to play a crucial role. It is a complicated progress, during which, epithelial cells lose their epithelial features and gain mesenchymal phenotypes, and epithelial cells become migratory and invasive.^[6,7]^ As one of transcription factors, snail homolog 1 (Snai1) is considered as one of the primary drivers of EMT and has been implicated in the EMT associated with tumor progression.^[8,9]^ The expression of snail has been disclosed to associate with invasion and metastasis as well as poor prognosis of many malignancies, such as gastric cancer,^[10]^ breast cancer,^[11]^ hepatocellular carcinoma,^[12]^ colorectal cancer^[13]^ and lung cancer.^[14]^ In recent years, a number of studies analyze the relationship between the snail expression and prognosis of patients with LC, but due to differences in research method, sample size and the study population, the findings of a single study are difficult to extend to the entire population and the obtained conclusions are inconsistent. The study conducted by Merikallio et al^[14]^ supported that the expression of snail was strongest in small cell lung cancer. Whereas, the prognostic role of snail is contradictory in NSCLC. Yanagawa et al^[15]^ indicated that lung adenocarcinoma patients with elevated snail expression had a significant reduction of survival time, while the high expression of snail was not a prognostic biomarker in lung squamous cell carcinoma. However, the study conducted by Sun et al^[16]^ indicated that snail was not a prognostic factor in lung adenocarcinoma. Given that the clinical results are controversial, we aim to systematically evaluate the prognostic role of snail in lung cancer patients.

## 2 Methods

### 2.1 Study registration

The protocol for this systematic review was registered on PROSPERO with registration number: CRD42018095191. This protocol follows the guidelines according to the Preferred Reporting Items for Systematic Reviews and Meta-Analyses Protocols (PRISMA-P) statement guidelines.^[17]^

#### 2.1.1 Data sources and search strategy

This study will perform a complete computer-based search of the PubMed, Embase, Web of Science, and the Cochrane Library databases for clinical trials up to the date of April 28, 2018. The strategy is created based on a discussion among all reviewers according to the Cochrane handbook guidelines. The following search terms will be used: lung neoplasm(s), lung cancer(s), lung tumor(s), lung carcinoma(s), pulmonary cancer(s), snail family transcription factors, snail, snail 1, prognosis, outcome, prognostic value, survival, and prognostic biomarker(s). The example search strategy in Table 1 will be used for PubMed. This search strategy will be modified and used for the other databases. The other strategies for Embase, Web of Science, and the Cochrane Library databases are shown in Supplement Tables 1–3. Besides, we will examine reference lists of all retrieved articles that may fulfill our eligibility requirements in order to avoid missing relevant studies.

**Table 1:**
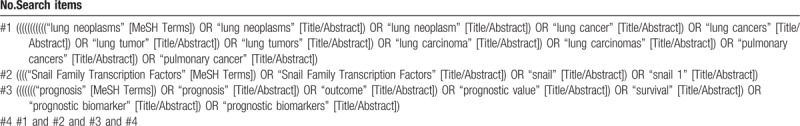
Search Strategy Used in PubMed.

### 2.2 Inclusion and exclusion criteria

The main inclusion criteria are as follows: Retrospective or prospective studies evaluating the prognostic relationship between the expression of snail and LC; the expression of snail in tissues detected by immunohistochemistry analysis; providing sufficient data to estimate the hazard ratios (HRs) for overall survival (OS) and progression-free survival (PFS)/relapse-free survival (RFS)/disease-free survival (DFS), along with their 95% confidence intervals (CIs) or p values; published in English.

The main exclusion criteria are as follows: Reviews, case reports, conference abstracts, specialist experience, comments and cell or animal studies. studies without enough data to pool the HRs; studies not focusing on the role of the snail expression on the clinicopathological features or prognoses in LC; not published in English.

### 2.3 Data extraction and quality assessment

#### 2.3.1 Selection of studies

**Figure 1:**
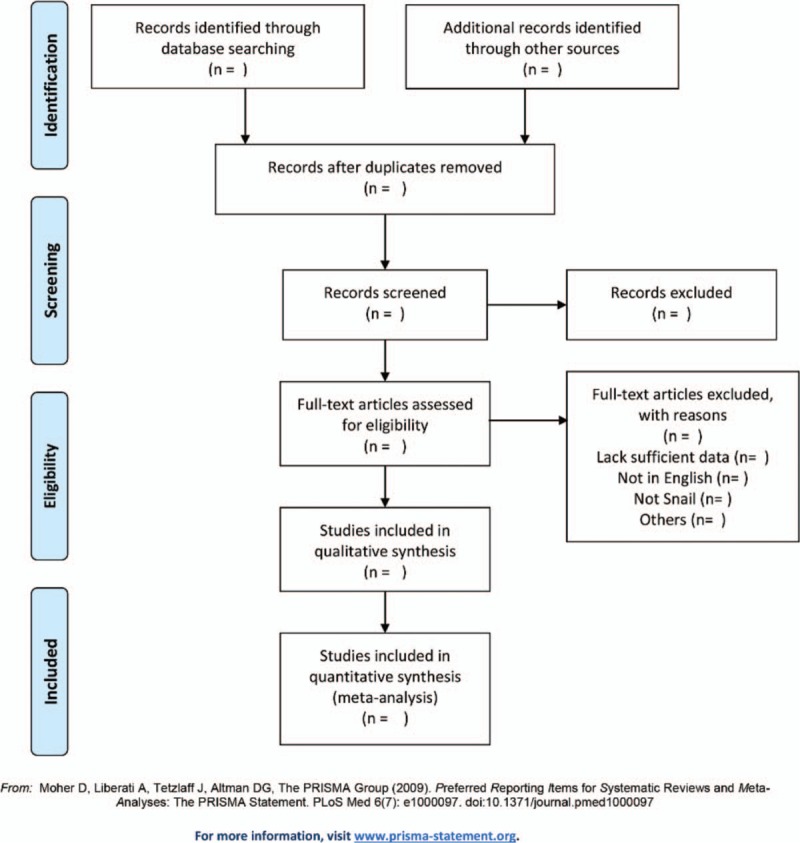
Flow diagram of studies identified.

All review authors have received training to ensure a good understanding of the purpose and process of the review. All identified studies will combine together in a single reference manager file created by Endnote X8 and duplicate studies will be removed using this software. The selection process will be conducted by 2 reviewers (MS and GD) independently. Initially, we will screen the titles, abstracts, and keywords of all retrieved records. The articles meeting inclusion criteria will be reviewed comprehensively by reading the full text. A table named “Reasons for excluded studies” will be used for recording excluded studies. We will resolve disagreements by consensus between the 2 reviews or by involving a third review author (ML). Using the PRISMA-compliant flowchart (http://www.prisma-statement.org), the screening and selection process of this study will be documented and summarized. The primary selection process is shown in a PRISMA flowchart (Fig. 1).

#### 2.3.2 Data extraction and management

Data extraction of the included studies will be performed by 2 independent authors (MS and GD). We will use a data extraction form (Excel) designed by all the authors in consensus to conduct the data extraction. The following information are extracted: name of the first author, publication time, country, number of patients, recruitment time, follow-up duration, analysis method, tumor type, clinicopathological features, antibody epitope, antibody company, method and score for its evaluation, cut-off value of snail overexpression, positive expression rate, HRs, and their 95% CIs. If HRs and their 95% CIs are not reported, we will extract them from Kaplan–Meier curves using the methods proposed by Tierney et al.^[18]^ Any disagreement will be solved by consensus or an arbiter (ML).

#### 2.3.3 Assessment of quality in included studies

The Newcastle–Ottawa Quality Assessment Scale (NOS) is employed to assess the quality of the selected studies. The NOS included 3 main aspects: selection, comparability, and outcome.^[19]^ A study with a score of at least 5 will be considered of high quality. Two independent authors (MS and DL) will use the criteria outlined in the NOS. Any disagreement will be resolved by discussion or by involving an arbiter (ML).

#### 2.3.4 Measures of prognosis

For prognostic outcomes including OS, PFS, RFS, DFS, data will be expressed as the HRs along with their 95% CIs or p values.

#### 2.3.5 Management of missing data

In several studies which some data are missing, we will consider why the data are missing (missing at random or not). We will try to contact the authors to request any inadequate and missing data of the included studies. If the data are still incomplete, available case analysis will be performed. And we will address the potential impact of missing data on the findings of the review in Section 3.

#### 2.3.6 Assessment of heterogeneity

The heterogeneity was assessed across all studies by Cochran's *Q* test and Higgins *I*^2^ method.^[20]^ When the result of a *Q*-test (*I*^2^ ≥ 50% or *P* < .05) indicating substantial heterogeneity, while *I*^2^<50% will be taken as evidence of no heterogeneity. In cases of substantial heterogeneity, we will explore the possible causes by sensitivity analysis and subgroup analyses.

#### 2.3.7 Assessment of publication biases

We will assess the publication bias with a funnel plot if the number of included studies is more than 10. Visual inspection of funnel plot, Egger's test, Begger's test^[21]^ are used to evaluate publication bias (*P* < .01 is considered statistically significant).

#### 2.3.8 Data synthesis

Meta-analysis will be performed using STATA software (version 14.0; Stata Corp, College Station, TX). The prognosis outcomes are explored using the HRs and the corresponding 95% CIs. If *I*^2^≥50%, the random-effect model will be used for data analysis, otherwise a fixed-effect model will be used for data analysis. Besides, the sensitivity analysis and subgroup analysis will be employed for exploring the causes of heterogeneity. All the p values are 2-side and *P* < .05 is considered statistically significant.

#### 2.3.9 Subgroup analysis

The prognosis outcomes mainly contain the OS and PFS/DFS/RFS. In cases of high heterogeneity, we will perform subgroup meta-analyses to determine the possible factors that may influence the results. The following subgroup analyses will be considered:

1. Different nationalities
2. Histology type of LC
3. Different statistical analyses.

#### 2.3.10 Sensitivity analysis

The sensitivity analysis will conduct using the “metaninf” STATA command (sequential exclusion of each individual study then pooled HRs) to examine the robustness of the pooled results.

## 3 Discussion

Cancer metastasis is the major cause for the poor survival of LC patients. The metastasis of LC is extremely complex processes, where multiple steps are involved.^[22]^ EMT is considered to be one of the major molecular mechanisms inducing tumor invasion, metastasis, and postoperative recurrence.^[23,24]^ It is an important cellular process, which occurs during cancer development and progression and has a crucial role in metastasis by enhancing the motility of tumor cells.^[25]^ The first step of EMT process is that epithelial cells lose cell–cell junctions and the epithelial marker E-cadherin.^[26]^ E-cadherin acts as a tumor suppressor inhibiting invasion and metastasis. While, downregulation of E-cadherin expression increases tumor cell motility and promotes invasion. The snail, which is zinc finger transcription factors, is overexpressed in epithelial cell lines and mainly repress expression of E-cadherin to reduce cell adhesion during the EMT.^[27,28]^ Therefore, snail is an important factor in regulating EMT and its high expression is related to the enhancement of LC invasion, metastasis, and progression.^[29]^

Several studies were conducted to explore the prognostic significance of snail in LC. Unfortunately, the results of these researches were controversial. A number of studies have shown that EMT transcription factor snail is closely related to the prognosis of patients with LC, but some individual studies show that there is no clear relationship between snail and the prognosis of patients with LC. Hence, we hope this review will provide more accurate and objective evidences of the relationship between the snail and the prognosis of patients with LC.

While, there are several limitations that need to be addressed in this review. Firstly, only studies published in English will be included, which may increase the bias of our study. Secondly, different nationalities, histology type of LC and different statistical analyses may run risk of heterogeneity. In addition, the methods and cut-off definitions for evaluating snail expression may be different.

The PRISMA-P checklist of the protocol is supplied in PRISMA-P checklist.

## Author contributions

**Data curation:** Meiling Shi, Guangtong Dong, Daorui Li.

**Supervision:** Peitong Zhang, Kaiwen Hu.

**Writing – original draft:** Meng Li, Xing Zhang.

**Writing – review & editing:** Meng Li.

## Supplementary Material

Supplemental Digital Content
