# Supplementary material for: Prognostic role of snail in lung cancer: Protocol for a systematic review
Source: Medicine (Baltimore). 2018 Jul 13;97(28):e11539. doi: 10.1097/MD.0000000000011539 (PMC6076196; doi:10.1097/MD.0000000000011539)
Supplement: Supplemental Digital Content [file medi-97-e11539-s001.docx]

Prognostic role of snail in lung cancer:

protocol for a systematic review

**Meng Li, PhD^a,b*^, Xing Zhang, PhD^a,b*^, Kaiwen Hu, PhD^c^, Meiling Shi^b^, Guangtong Dong^b^, Daorui Li, PhD^b^, Peitong Zhang, PhD^b*^**

^a^Beijing University of Chinese Medicine, Beijing, China, ^b^Department of Oncology, Guang’anmen Hospital, China Academy of Chinese Medical Sciences, Beijing, China, ^c^Department of Oncology, Dongfang Hospital, Beijing University of Chinese Medicine, Beijing, China.

*Correspondence should be addressed to:

Peitong Zhang: peitong_zhang@163.com

Supplementary Table 1. Search Strategy Used in EMBASE

| No. | Search items |
| --- | --- |
| #1 | 'lung tumor'/exp OR 'lung tumor':ti,ab,kw OR 'lung tumors':ti,ab,kw OR 'lung cancer':ti,ab,kw OR 'lung cancers':ti,ab,kw OR 'lung carcinoma':ti,ab,kw OR 'lung carcinomas':ti,ab,kw OR 'lung neoplasm':ti,ab,kw OR 'lung neoplasms':ti,ab,kw |
| #2 | 'snail family transcription factors'/exp OR 'snail'/exp OR 'snail 1' |
| #3 | 'prognosis'/exp OR 'prognosis':ti,ab,kw OR 'outcome':ti,ab,kw OR 'prognostic value':ti,ab,kw OR 'survival':ti,ab,kw OR 'prognostic biomarker':ti,ab,kw OR 'prognostic biomarkers':ti,ab,kw |
| #4 | #1 and #2 and #3 and #4 |

Supplementary Table 2. Search Strategy Used in Web of Science

| No. | Search items |
| --- | --- |
| #1 | (lung neoplasm*) |
| #2 | (lung cancer*) |
| #3 | (lung tumor*) |
| #4 | (lung carcinoma*) |
| #5 | (pulmonary cancer*) |
| #6 | #1 OR #2 OR #3 OR #4 OR #5 |
| #7 | (Snail*) |
| #8 | (Snail Family Transcription Factor*) |
| #9 | (Snail 1) |
| #10 | #7 OR #8 OR #9 |
| #11 | (prognosis*) |
| #12 | (outcome*) |
| #13 | (prognostic value*) |
| #14 | (survival*) |
| #15 | (prognostic biomarker*) |
| #16 | #11 OR #12 OR #13 OR #14 OR #15 |
| #17 | #6 AND #10 AND #16 |

Supplementary Table 3. Search strategy used in the Cochrane Library

| **No.** | **Search items** |
| --- | --- |
| #1 | MeSH descriptor Lung Neoplasms explode all trees |
| #2 | (lung neoplasm?) |
| #3 | (lung cancer?) |
| #4 | (lung tumor?) |
| #5 | (lung carcinoma?) |
| #6 | (pulmonary cancer?) |
| #7 | #1 or #2 or #3 or #4 or #5 or #6 |
| #8 | MeSH descriptor Snails explode all trees |
| #9 | (Snail?) |
| #10 | MeSH descriptor Snail Family Transcription Factors explode all trees |
| #11 | (Snail Family Transcription Factor?) |
| #12 | (Snail 1) |
| #13 | #8 or #9 or #10 or #11 or #12 |
| #14 | MeSH descriptor prognosis explode all trees |
| #15 | (prognosis*) |
| #16 | (outcome?) |
| #17 | (prognostic value?) |
| #18 | (survival?) |
| #19 | (prognostic biomarker?) |
| #20 | #14 or #15 or #16 or #17#18 or #19 |
| #21 | #7 and #13 and #20 |
